# Supplementary material for: Obesity, the Adipose Organ and Cancer in Humans: Association or Causation?
Source: Biomedicines. 2023 Apr 28;11(5):1319. doi: 10.3390/biomedicines11051319 (PMC10215824; doi:10.3390/biomedicines11051319)
Supplement: Supplementary file 1 [file biomedicines-11-01319-s001.zip › biomedicines-2251838-supplementary.pdf]

Endometrial and ovarian cancer

Among women, endometrial cancer is more strongly associated with obesity than any other cancer type [39]. The RR was 2.89 (CI 2.62-3.18) with a 10 kg/m^2 increase in BMI [40] or 1.59 (CI 1.50-1.68) with a 5 kg/m^2 increase in BMI [28]. Compared with women with a normal weight, the RR of mortality for women with moderate obesity (BMI 35-39.99 Kg/m^2) is 2.53, and for severe obesity (≥ 40 Kg/m^2) is 6.25 [27]. Moreover, obesity is also associated with a worse outcome [41].

Although the association is not as strong as with endometrial cancer, obesity is also related to the risk of developing other gynaecological cancers, particularly ovarian cancer [42]. The RR was 1.14 (CI 1.03-1.27) with a 10 kg/m^2 increase in BMI or 1.03 (CI 0.99-1.08) with a 5 kg/m^2 increase in BMI [28]. It has been hypothesised that visceral fat is more linked to the development of ovarian cancer than BMI [43].

Esophageal adenocarcinoma and gastric cardia cancer

There are several risk factors for esophageal adenocarcinoma (EAC) and gastric cardia cancer and above all gastroesophageal reflux disease (GERD) and obesity. The RR for EAC occurrence is 1.52 (CI 1.33-1.74) for each 5kg/m^2 increase in BMI in men and 1.51 in females [28] but abdominal obesity, in particular, is related to Barrett's oesophagus, and to EAC (Odds Ratio 2.51). Obesity is not linked to EAC only due to an anatomic matter, since abdominal obesity is associated with Barrett's oesophagus even after adjusting for GERD, and obesity is associated with EAC even in individuals without symptoms of GERD. A multicenter study demonstrated an association of visceral adiposity, measured by computed tomography, with esophageal and junctional adenocarcinomas [44]). However, we demonstrated that overweight patients operated on for EAC survive longer than normal-weight patients [11].

Breast cancer

Breast cancer is associated with a greater risk of developing in postmenopausal women with obesity, especially for BMI > 35.0 kg/m^2 [45]. In epidemiological studies, the relative risk (RR) was 1.40 (CI 1.31-1.49) with a 10 kg/m^2 increase in BMI [40] or 1.12 (CI 1.08-1.16) with a 5 kg/m^2 increase in BMI [28]. Although obesity is a risk factor for postmenopausal hormone receptor-positive breast cancer [45,46], it is inversely associated with progesterone receptor (PR) and estrogen receptor (ER)-positive premenopausal breast cancer [47]. Furthermore, increased BMI in adolescence decreases the risk of all ER/PR/HER2-defined subtypes of premenopausal breast cancer and this advantage

was higher in women who consistently have high BMI during their premenopausal years [48]. It seems that the protective effects of hormones prevail during premenopause in patients with obesity. Inversely, in postmenopause, the inflammation combined with a higher aromatase activity increases estrogen production and insulin-resistance. These mechanisms, accompanied by higher levels of biologically available estradiol, increase breast cancer risk observed in epidemiological studies [49,50].

Liver cancer (hepatocellular carcinoma)

In western countries, a large amount of hepatocellular carcinoma (HCC) develop from NAFLD or, according to the new definition, metabolic associated fatty liver disease (MAFLD) [51], which is one of the main comorbidities in patients with obesity and type 2 diabetes mellitus (T2DM) [52,53]. Therefore, obesity is related to increased cirrhosis, HCC and risk of death, particularly for male patients with moderate obesity: RR 4.52 (CI 2.94–6.94) [27]. Nevertheless, patients with obesity and cirrhosis have lower mortality than normal weight patients with cirrhosis and markers of fibrosis such as FIB-4 displayed an inverse correlation with BMI [54,55].

Kidney cancer (renal cell carcinoma)

Over 40% of RCC seem to be associated with obesity assessed on the basis of BMI. The RR was 1.24 (CI 1.15-1.34) for men and 1.34 (CI 1.25-1.43) for women with a 5 kg/m^2 increase in BMI [28]. The RR of the highest BMI category versus normal BMI was 1.8 (CI 1.7-1.9), according to a strong body of evidence [29]. The results of a large, prospective study revealed that weight gain until 50 years of age strongly correlated with the incidence of RCC, whereas weight gain after 50 years was not so strongly related to RCC [56]. The Metabolic Syndrome and Cancer Project (Me-Can) showed that increased levels of BMI, blood pressure, glucose and triglycerides were associated with increased risk of RCC among men, while high BMI was most important in women [57].

Colorectal cancer

The relationship between obesity and colorectal cancer (CRC) development has been evaluated in several epidemiological studies [27-29]. The RR was 1.24 (CI 1.20-1.28) for men and 1.09 (CI 1.05-1.13) for women with a 5 kg/m^2 increase in BMI and the association was stronger in men than in women ($p<0.0001$) [28,58]. The RR for CRC was higher in patients with obesity compared with normal-weight patients [59]. In a large meta-analysis higher BMI was significantly associated with

more favourable CRC outcomes, even though higher BMI/obesity is an established determinant for the development of CRC [13].

Gallbladder cancer

The impact of obesity on the incidence of gallbladder cancer is more pronounced in females (men 11%, women 42%) and the RR of death from gallbladder cancer for a BMI of at least 30.0 kg/m^2 was 2.44 [27]. The RR of the highest BMI category versus normal BMI was 1.3 (CI 1.2-1.4), according to a strong body of evidence [29]. Beyond molecular mechanisms, obesity may operate indirectly by increasing the risk of gallstones, which, in turn, increase the risk of gallbladder cancer [60].

Pancreatic cancer

The RR for pancreatic cancer occurrence was 1.16 (CI 1.05-1.28) for each 5kg/m^2 increase in BMI in men and 1.10 (CI 1.02-1.09) in women [61]. According to Renehan et al [28] the RR was lower for men. The RR of the highest BMI category versus normal BMI was 1.5 (CI 1.2-1.8) [29].

Prostate cancer

The relationship between BMI and prostate cancer was much discussed [28,62]. Cancer-related mortality for male patients with moderate obesity showed a RR of 1.34 (CI 0.98–1.83) [27]. While a high BMI was positively related with non-metastatic high-grade prostate cancer, it was inversely related with the risk of low-grade tumour. It can be suggested that the pathogenesis of high-grade disease may be associated with low grade inflammation of obesity. For instance, the leptin receptor G2548A genetic variant is statistically significantly associated with an increased risk of prostate cancer. Moreover, patients with high-volume prostate cancer had higher serum leptin levels than those with low-volume prostate cancer. Interestingly, an inverse association between BMI and prostate-specific antigen (PSA), used for prostate cancer screening, was demonstrated [62,63].

Thyroid cancer

The RR for thyroid cancer occurrence was 1.33 (CI 1.04-1.70) for each 5kg/m^2 increase in BMI in men, with lower RR in women [28]. A statistically significant 25% greater risk of thyroid cancer was found in individuals with overweight and a 55% greater thyroid cancer risk in individuals with

obesity as compared with normal-weight people. Each 5-unit increase in BMI and 0.1-unit increase in waist-to-hip ratio were associated with 30% and 14% greater risks of thyroid cancer, respectively. Moreover, both general and abdominal adiposity were positively associated with thyroid cancer [64]. Interestingly, with regard to histologic type, obesity was significantly positively related to papillary, follicular and anaplastic thyroid cancers, while there was an inverse association with medullary thyroid cancer [64-66].

Multiple myeloma

Multiple myeloma is the second most common hematologic malignancy in the United States. The RR for multiple myeloma occurrence was 1.11 (CI 1.05-1.18) for each 5 kg/m^2 increase in BMI in men with very similar values for women [28]. High BMI is positively associated with poor outcomes [30] and the incidence of multiple myeloma significantly increased in young adults (25–49 years) in a stepwise manner in successively younger generations [67].

Mouth, pharynx and larynx cancer

Excess body fatness is a strong risk factor for cancers of the mouth, pharynx and larynx [68], in particular RR was: 0.81 (CI 0.74–0.89) for oral cavity cancers for each 5kg/m^2 increase in BMI; 0.94 (CI 0.57–1.56) for laryngeal cancer in patients with obesity compared to normal weight patients; 0.31 (CI 0.08–1.11) for oropharyngolaryngeal cancers for patients with BMI ≥23.2 kg/m^2 vs patients with BMI ≤18.9 kg/m^2 and 0.87 (0.51–1.50) for each 5kg/m^2 increase in BMI for upper aerodigestive tract cancers for patients with 23.5kg/m^2 < BMI < 35kg/m^2. However, these data were not adjusted for smoking, a strong potential confounder. In fact, tobacco smokers tend to have less healthy diets, more sedentary ways of life, and lower body weight than non-smokers. Therefore, a central task in assessing the results of dietary studies is to evaluate the degree to which observed associations in smokers may be due to residual confounding by cigarette smoking; that is, not a direct result of the dietary exposure examined. When smoking or drinking alcohol (another risk factor for these types of cancer) are taken into consideration, controversial results arise and oral cavity, pharynx and larynx cancer risk ends up being associated with a lower BMI [69,70]. Therefore, further studies are needed in order to avoid potential reverse causality.

Lung cancer (non-small-cell lung cancer)

Obesity was considered a positive prognostic factor for non-small-cell lung cancer (NSCLC) [71] and it was associated with increased lung cancer survival [72]. Furthermore, patients with obesity respond better to immunologic therapies such as pembrolizumab [73] or atezolizumab [74]. Barbi et al. showed that visceral fat area normalised to total fat area (visceral fat index), was not correlated to BMI and it was associated with decreased recurrence-free and overall survival in early stage NSCLC [75], whereas high BMI was not. An important confounder of obesity's contribution to lung cancer outcomes may be not only the limitation of using BMI but also the frequent use of metformin in these patients [76]. In fact, metformin reduces tobacco carcinogen-induced lung carcinogenesis and its use is linked to improved survival in patients with lung cancer.
